# Association of Radiologic Findings with Mortality in Patients with Avian Influenza H7N9 Pneumonia

**DOI:** 10.1371/journal.pone.0093885

**Published:** 2014-04-04

**Authors:** Feng Feng, Yebin Jiang, Min Yuan, Jie Shen, Huabin Yin, Daoying Geng, Jianrong Xu, Yanqing Hua, Jingyun Shi, Yuxin Shi, Zhiyong Zhang

**Affiliations:** 1 Department of Radiology, Shanghai Public Health Clinical Center, Fudan University, Shanghai, China; 2 Department of Radiology, The Fifth People's Hospital of Shanghai, Fudan University, Shanghai, China; 3 Department of Radiology, Huashan Hospital, Fudan University, Shanghai, China; 4 Department of Radiology, Renji Hospital, Shanghai Jiaotong University, Shanghai, China; 5 Department of Radiology, Huadong Hospital, Fudan University, Shanghai, China; 6 Department of Radiology, Shanghai Pulmonary Hospital, Tongji University, Shanghai, China; 7 Cancer Center, University of Michigan, Ann Arbor, Michigan, United States of America; 8 Department of Radiology, Nantong Tumor Hospital, Nantong University, Nantong, China; University of Calgary & ProvLab Alberta, Canada

## Abstract

**Background:**

The novel H7N9 virus causes severe illness, including pneumonia and acute respiratory distress syndrome, with high rates of mortality. We investigated the association of initial radiologic characteristics obtained at admission with clinical outcomes in patients with avian influenza H7N9 pneumonia.

**Methods:**

Demographics, comorbidities, clinical findings, radiologic appearance and scores of the affected lung parenchyma were compared between survivor group (n = 15) and mortality group (n = 7). Two radiologic scores were calculated, one using chest radiography and one using CT. Follow-up CT scans at discharge were analyzed in 12 patients of the survival group.

**Results:**

All the patients in mortality group developed acute respiratory distress syndrome and required mechanical ventilation, while in the survival group 33% (5/15) developed acute respiratory distress syndrome (*P*<0.05) and 27% (4/15) required mechanical ventilation (*P*<0.05). The mean radiographic and CT scores of the mortality group were 50% higher compared to the survival group (*P*<0.05). ROC analysis revealed an area under curve of 0.738 for the radiographic score with an optimal cutoff value of a score of 19 for prediction of mortality, with a sensitivity of 71% and a specificity of 67%, and an area under curve of 0.833 for the CT score with an optimal cutoff value of a CT score of 21 for prediction of mortality, with a sensitivity of 86% and a specificity of 73%. The mean CT score of the affected lung parenchyma at discharge was 30% lower than the initial CT examination (*P*<0.05).

**Conclusion:**

High initial radiologic score is associated with mortality in patients with avian influenza H7N9 pneumonia.

## Introduction

Influenza is a highly contagious disease of global importance. The virus infects a variety of animals including birds, and infects humans. Three subtypes of influenza A were previously reported to infect humans, i.e., H1N1, H1N2, and H3N2 [1]. During the past few years, several subtypes of avian influenza A have been shown to cross the species barrier and infect humans. In the spring of 2013, a novel avian-origin influenza A virus, H7N9, emerged and spread among humans in China. The first documented case of human infection with the influenza H7N9 virus occurred in Shanghai in March [2]. As of May 9, the World Health Organization (WHO) reported 131 laboratory-confirmed cases, including 32 deaths [3]. Of the 111 patients with the H7N9 virus infection has been reported recently, 76.6% were admitted to an intensive care unit (ICU) [4]. On admission, 108 patients (97.3%) had findings consistent with pneumonia. The clinical features, blood cell counts, and laboratory and biochemical findings in patients with H7N9 virus infection have been described [5]–[7]. Patients usually presented with fever and cough, and with early sputum production. The illness progressed rapidly to severe pneumonia, with moderate-to-severe acute respiratory distress syndrome [5].

During the outbreak of avian flu, imaging studies provided important information for diagnosis, management, and control of H7N9 infection [6]. On chest radiographs or computed tomographic (CT) scans, bilateral ground glass opacities and consolidation were the typical radiologic findings [4], [8].

Because H7N9 virus infection is potentially fatal disease and extremely contagious with mortality rate of 27% [4], it is important during the current influenza outbreak to identify initial imaging findings as potential risk stratification to help triage patient, guide treatment, and monitor disease progression and treatment response. We therefore investigated the association of initial chest radiographic findings and CT characteristics obtained at admission with clinical outcomes in patients with avian influenza H7N9 pneumonia.

## Methods

### Patients

This study was approved by the Institutional Review Boards of Shanghai Public Health Clinical Center, the Fifth People's Hospital of Shanghai, Huashan Hospital, Renji Hospital, Huadong Hospital, and Shanghai Pulmonary Hospital. The requirement of informed consent was waived. Between March 2013 and May 2013, 22 patients who fulfilled the clinical criteria for H7N9 influenza infection established by the National Centers for Disease Control and Prevention (CDC) were hospitalized in the above 6 different institutions. Diagnosis of H7N9 infection was made by positive test for H7N9 viral RNA, in throat-swab specimens collected from patients by real-time reverse transcription polymerase chain reaction (RT-PCR) using standard RT-PCR protocol at Shanghai CDC. All patients were in-hospital and had undergone chest radiographs and CT scans. Two radiologic scores were calculated, one using chest radiography and one using CT.

The 22 patients were treated with oral oseltamivir 75 mg, twice daily. Patients were also given antibiotics, included moxifloxacin, sulbactam and cefoperazone, levofloxacin, meropenem, piperacillin, imipenem, and cilastatin, when there was positive test in blood and/or throat-swab for specific bacterial infection. Some patients also received glucocorticoid, or intravenous immunoglobulin administration.

### Chest radiographs

Chest radiographs were obtained using conventional radiography with posteroanterior projection, or portable computed radiography at bedside with anteroposterior projection.

### CT scans

The types of scanners and protocols used for the CT scans varied since they were from 6 different institutions. The technical parameters included 0.75 mm or 1 mm collimation at 5–7 mm intervals. Follow-up scans were obtained at discharge from 12 patients. All examinations were performed with the patient in the supine position and with breath-holding following inspiration, without administration of contrast material.

Images were obtained with both mediastinal (width 350–450 HU; level 20–40 HU) and parenchymal (width 1200–1600 HU; level −500 to−700 HU) window settings.

### Imaging evaluation

Three chest radiologists reviewed the images independently initially, with a final finding reached by consensus when there was a discrepancy. They were blinded to the clinical information or clinical progress of the patients, except for the knowledge that these were cases of H7N9 virus infection.

Chest radiographic and CT findings included ground glass opacity, consolidation, and nodular opacities. Ground glass opacities were defined as hazy areas of increased opacity or attenuation without concealing the underlying vessels. Consolidation was defined as homogeneous opacification of the parenchyma obscuring the underlying vessels. Nodular opacities were defined as focal round opacities (diameter <2 cm). The presence of lymphadenopathy defined as a lymph node ≥1 cm in short-axis diameter.

**Figure 1:**
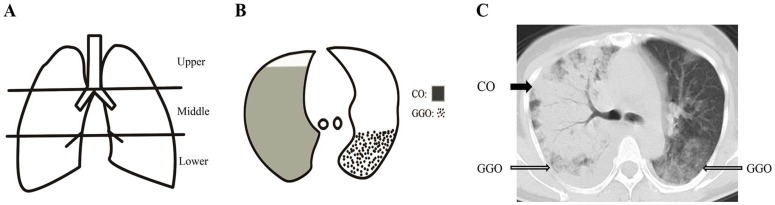
The schematic of CT scoring system. Schematic coronal representations of zones, extent of ground-glass opacity and consolidation on an axial image, and a sample scoring on an axial CT image. Schematic coronal representation of zones (A) evaluated in the lungs of patients with H7N9 infection shows upper zones which are above the carina, middle zones between the carina and the inferior pulmonary vein, and lower zones which are below the inferior pulmonary vein. Schematic representation of the extent of ground-glass opacity (GGO) and consolidation (CO) on an axial image (B) which were determined using the modified Grieser system. Points from all zones were added for a cumulative score, with value ranging from 0 to 72. A sample scoring on an axial CT image (C) of a 52-year-old woman from mortality group demonstrates a total score of 18, calculated as 3 (consolidation, solid arrow) ×4 (>75% distribution in middle zone of the right lung) +2 (ground-glass opacity, open arrow) ×1 (<25% distribution in middle zone of the right lung) +2 (ground-glass opacity, open arrow) ×2 (25–50% distribution in middle zone of the left lung).

The extent of involvement of each abnormality was assessed independently for each of 3 zones: upper (above the carina), middle (below the carina and above the inferior pulmonary vein), and lower (below the inferior pulmonary vein). The location of the lesion was defined as peripheral if it was in the outer one-third of the lung, or as central otherwise. The radiographic and CT findings were graded on a 3-point scale: 1 as normal attenuation, 2 as ground-glass attenuation, and 3 as consolidation. The CT scans were scored on the axial images. The ground-glass attenuation and consolidation areas were determined using the method described by Grieser et al [9]. The Hounsfield Units (HU) of normal attenuation, ground-glass attenuation and consolidation areas were measured. Each lung zone, with a total of six lung zones in each patient, was assigned a following scale according to distribution of the affected lung parenchyma using a method modified from a previously described protocol [10]: 0 as normal, 1 as <25% abnormality, 2 as 25–50% abnormality, 3 as 50–75% abnormality, and 4 as >75% abnormality. The four-point scale of the lung parenchyma distribution was then multiplied by the radiologic scale described above (Figure 1). Points from all zones were added for a final total cumulative score, with value ranging from 0 to 72.

### Statistical analysis

All data were expressed as mean ± standard deviation (SD), or median and interquartile range (IQR) for the continuous variables and as a number of individuals with percentage in each group for the categoric variables. The comparison of continuous variables between groups was performed using the Mann-Whitney U test. Frequencies of categoric variables were compared by the Fisher exact test. A receiver operating characteristic curve was plotted to determine the appropriate cut-off value with maximum sensitivity and specificity for the endpoint of death. All analyses were considered significant at p values of less than 0.05 (two-tailed).

## Results

The 22 patients included 5 women and 17 men, with median age of 67 years (interquartile range, IQR, 56–75 years). Of the 22 patients, 20 (91%) were admitted to an intensive care unit (ICU), including 14 directly admitted to ICU and 6 transferred to ICU during hospitalization. The main adverse outcome measure was in-hospital death. As of July 10, a total of 7 patients had died in hospital, and 15 patients were discharged in recovered condition. The median time from the onset of illness to death was 18 days (IQR, 12 to 54 days). All the patients were devided into mortality (n = 7) and survival (n = 15) groups.

Table 1 shows the comparison of patient age, sex, comorbid illnesses, outcomes as resulting in required mechanical ventilation or developing acute respiratory distress syndrome, and clinical symptoms, between mortality and survival groups. The mortality rate in this study was 32% (7 of 22 patients). There were no significant differences between the two groups with respect to patient age, sex, comorbidities, and clinical manifestations. All the patients in mortality group developed acute respiratory distress syndrome and all of them required mechanical ventilation, while in survival group 33% (5/15) developed acute respiratory distress syndrome (*P* = 0.004) and 27% (4/15) required mechanical ventilation (*P* = 0.005). Only two patients in mortality group required invasive mechanical ventilation, while others required non-invasive mechanical ventilation.

**Table 1:** Demorgraphics, comorbidities, and clinical presentations in mortality and survival groups.

Variable	All patients	Survival Group	Mortality Group
	N = 22	N = 15	N = 7
Age, median(*IQR*), years	67(56–75)	67(56–78)	67(52–86)
Sex: men, women	17,5	12,3	5,2
Exposure to live poultry	2(9)	1(7)	1(14)
Coexisting medical condition			
Any	15(68)	10(67)	5(71)
Hypertension	9(41)	7(47)	2(29)
Diabetes	4(18)	3(20)	1(14)
Coronary heart disease	4(18)	3(20)	1(14)
Chronic obstructive pulmonary disease	2(9)	2(13)	0
Brest cancer	1(5)	1(7)	0
Hepatitis B infection	1(5)	0	1(14)
Outcome			
Acute respiratory distress syndrome	12(55)	5(33)	7(100) **
Requiring mechanical ventilation	11(50)	4(27)	7(100) **
Symptoms			
Fever >38°C	22(100)	15(100)	7(100)
Cough	20(91)	14(93)	6(86)
Dyspnea	16(73)	10(67)	6(86)
Expectoration	4(18)	3(20)	1(14)

**P<0.01 versus survival group.

Percentages are in parentheses, unless indicated as IQR (interquartile range).

Table 2 compares the frequency of chest radiographic findings, distribution patterns, and chest radiographic score of the affected lung parenchyma between the survival group and the mortality group. The predominant chest radiographic findings at presentation consisted of a bilateral mixed pattern of ground glass opacity and areas of consolidation in 18 patients (82%), air bronchograms in 11 patients (50%), and both central and peripheral distributions in 21 patients (95%). A small pleural effusion was found in 9 patients (41%). The mediastinal lymphadenopathies, pneumothorax and pneumomediastinum were not seen on chest radiographs.

**Table 2:** Chest radiographic features in mortality and survival groups.

Chest radiographic features	All patients	Survival Group	Mortality Group
	N = 22	N = 15	N = 7
Symptom onset before chest radiograph, median(*IQR*), days	7(4–8)	7(3–9)	6(4–7)
GGO	2(9)	2(13)	0
Consolidation	1(5)	0	1(14)
Both of GGO and consolidation	19(86)	13(87)	6(86)
Radiographic score, median(*IQR*)	18(16–24)	18(11–24)	27(18–31) *
Air bronchogram	11(50)	7(47)	4(57)
Nodular opacities	0	0	0
Lymphadenopathy	0	0	0
Pleural effusions	9(41)	6(40)	3(43)
Unilateral	5(23)	3(20)	2(29)
Bilateral	4(18)	3(20)	1(14)
Anatomic sides involved			
Unilateral	3(14)	2(13)	1(14)
Bilateral	19(86)	13(87)	6(86)
Predominant distribution			
Central	0	0	0
Peripheral	1(5)	1(7)	0
Both central and peripheral	21(95)	14(93)	7(100)
Involved zone			
Upper	17(77)	11(73)	6(86)
Middle	22(100)	15(100)	7(100)
Lower	22(100)	15(100)	7(100)

*P<0.05 versus survival group.

Percentages are in parentheses, unless indicated as IQR (interquartile range).

GGO: ground grass opacity.

**Figure 2:**
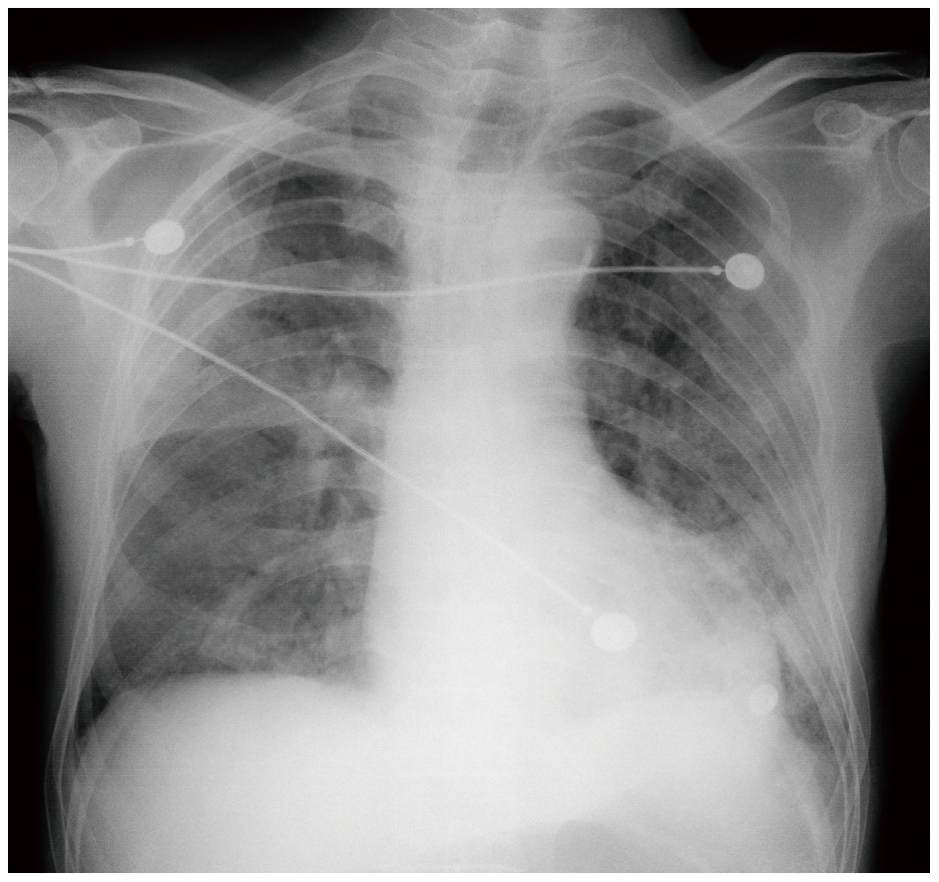
A 74 year old male patient with H7N9 pneumonia from mortality group. Chest radiograph with score of 28 shows bilateral patchy consolidations and ground glass opacities.

**Figure 3:**
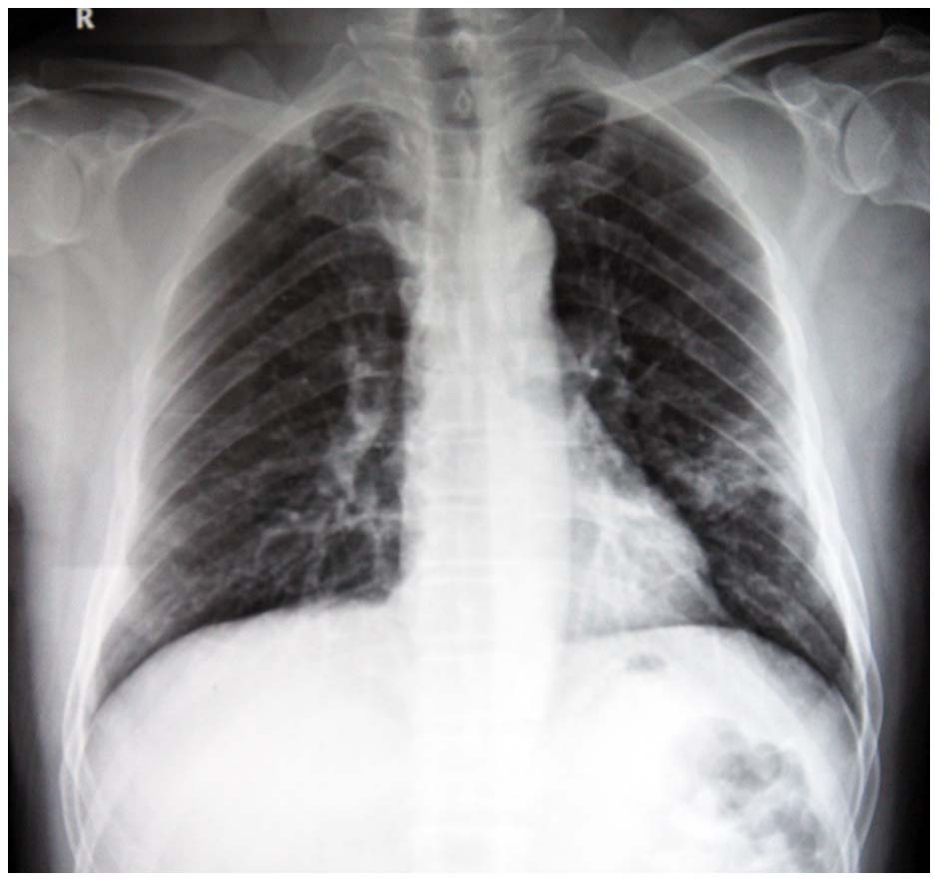
A 67 year old male patient with H7N9 pneumonia from survival group. Chest radiograph with score of 4 shows patchy consolidations and ground glass opacities in the left middle and lower zones.

**Figure 4:**
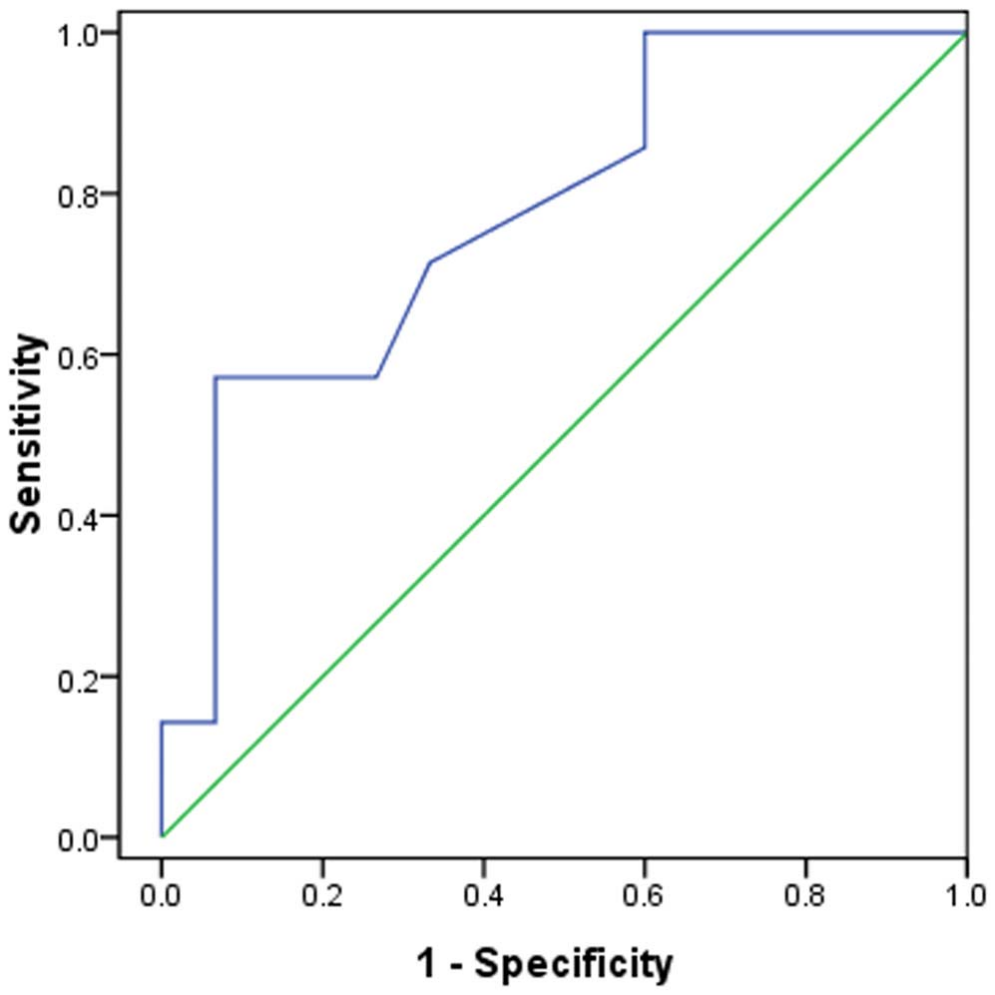
ROC analysis of the chest radiographic score for prediction of mortality. N = 22; AUC  = 0.738.

The mean chest radiographic score of the mortality group (Figure 2) was 50% higher compared to survival group (Figure 3) (*P* = 0.035). In the receiver operating characteristic curve analysis (Figure 4), an optimal cutoff value of a chest radiographic score of 19 had a sensitivity of 71% and a specificity of 67% for the prediction of mortality. The area under the receiver operating characteristic curve was 0.738 (95% confidence interval: 0.568, 0.985). There was no statistically significant difference in the frequency and distribution of the chest radiographic findings between the survival and mortality groups.

Table 3 compares the frequency of CT findings, distribution patterns, and CT score of the affected lung parenchyma between the survival group and the mortality group. The predominant CT findings at presentation consisted of a bilateral mixed pattern of ground glass opacity and areas of consolidation in 20 patients (91%), air bronchograms in 15 patients (68%), and both central and peripheral distributions in 21 patients (95%). A small pleural effusion was found in 12 patients (55%). The mediastinal lymphadenopathies were observed in 2 patients (9%) with coexistent chronic obstructive pulmonary disease. Pneumothorax and pneumomediastinum were not seen on CT.

**Table 3:** CT features in mortality and survival groups.

CT features	All patients	Survival Group	Mortality Group
	N = 22	N = 15	N = 7
Symptom onset before CT, median*(IQR)*, days	7(3–9)	7(4–9)	6(4–8)
GGO	1(5)	1(7)	0
Consolidation	1(5)	0	1(14)
Both of GGO and consolidation	20(91)	14(93)	6(86)
CT score, median(*IQR*)	21(15–24)	18(11–23)	27(21–33) *
Air bronchogram	15(68)	10(67)	5(71)
Nodular opacities	2(9)	2(13)	0
Lymphadenopathy	2(9)	2(13)	0
Pleural effusions	12(55)	8(53)	4(57)
Unilateral	6(27)	4(27)	2(29)
Bilateral	6(27)	4(27)	2(29)
Anatomic sides involved			
Unilateral	2(9)	1(7)	1(14)
Bilateral	20(91)	14(93)	6(86)
Predominant distribution			
Central	0	0	0
Peripheral	1(5)	1(7)	0
Both central and peripheral	21(95)	14(93)	7(100)
Involved zone			
Upper	18(82)	12(80)	6(86)
Middle	22(100)	15(100)	7(100)
Lower	22(100)	15(100)	7(100)

*P<0.05 versus survival group.

Percentages are in parentheses, unless indicated as IQR (interquartile range).

GGO: ground grass opacity.

**Figure 5:**
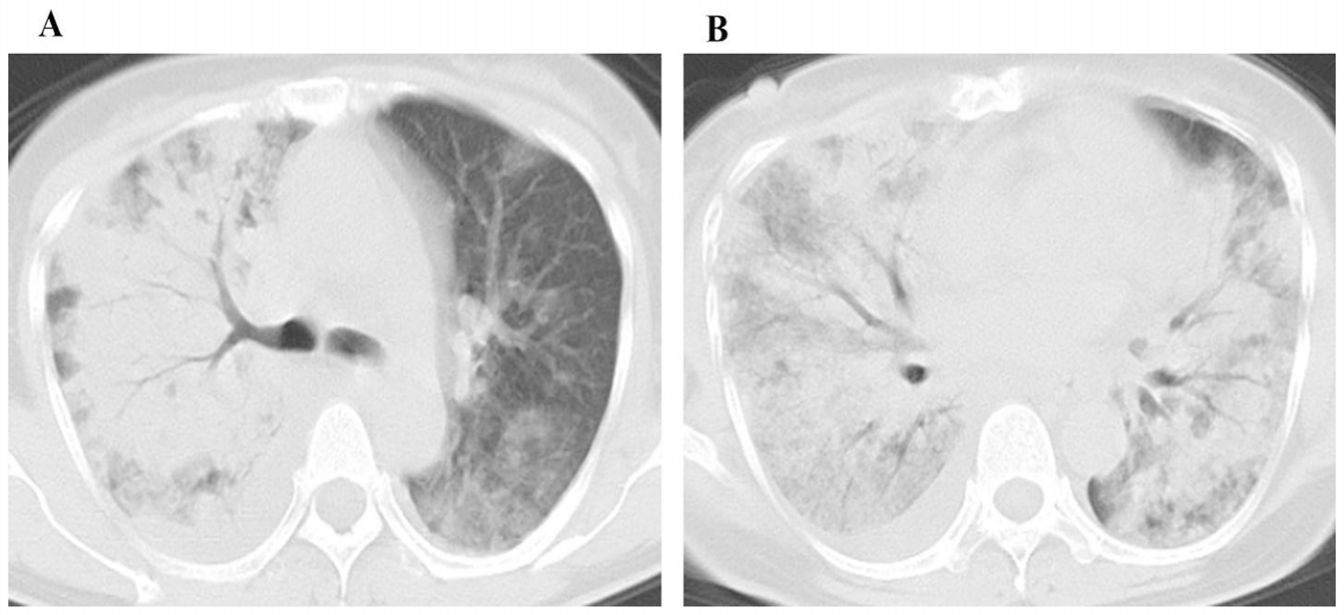
A 52 year old female patient with H7N9 pneumonia from mortality group. CT images with score of 38 show ground glass opacity and consolidation in the middle (A) and the lower zones (B). There is a small amount of right pleural effusion.

**Figure 6:**
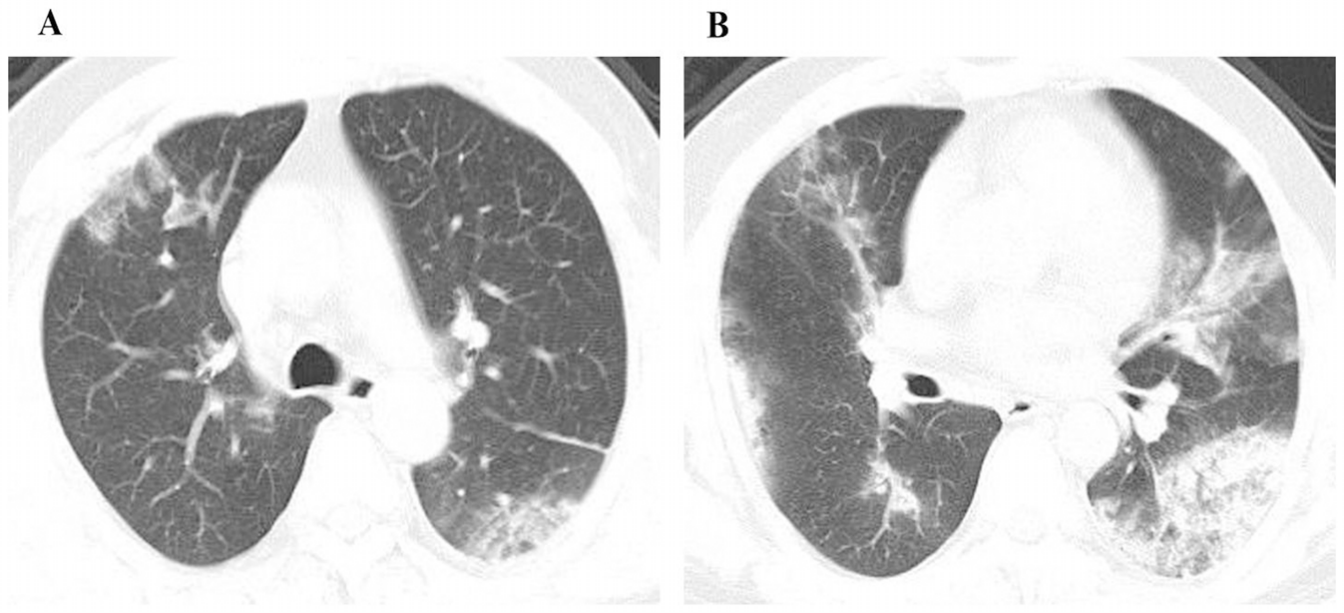
A 53 year old male patient with H7N9 pneumonia from survival group. CT images with CT score of 18 show ground glass opacity mostly in the upper zones (A) and consolidation in the lower zones (B).

**Figure 7:**
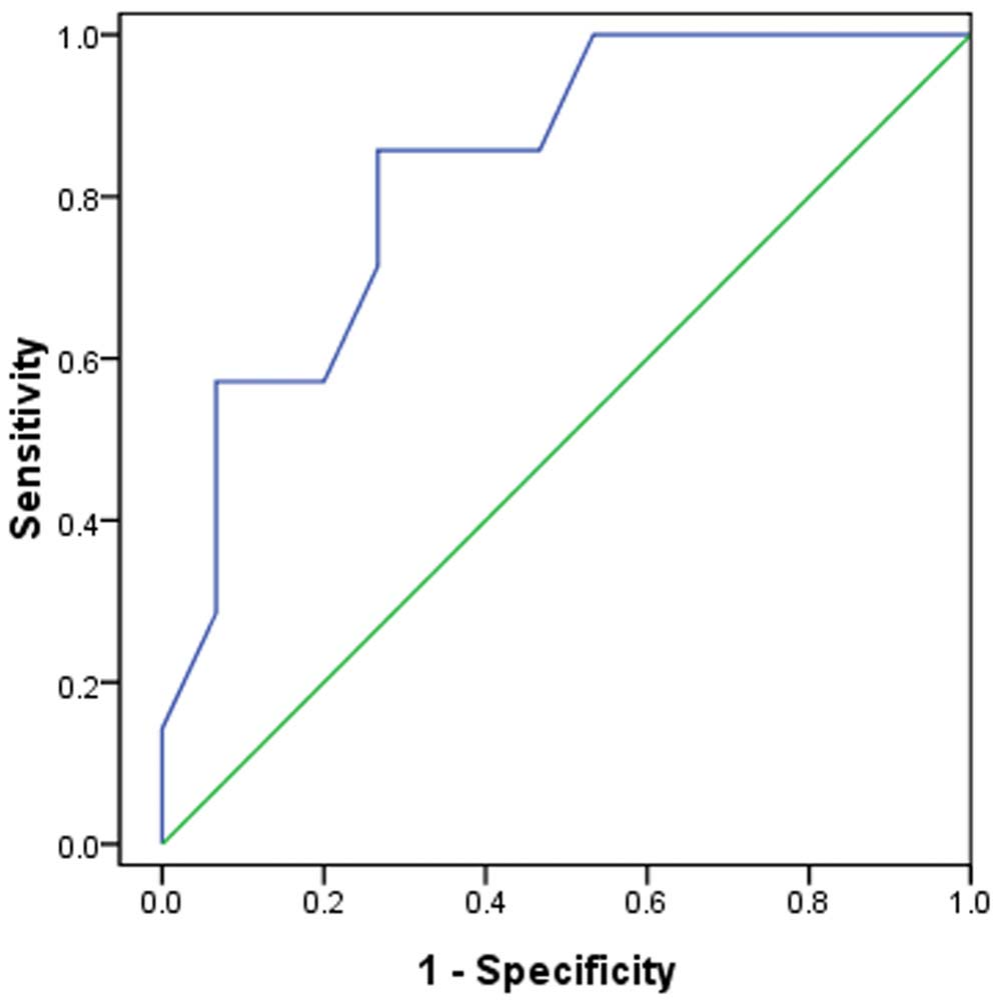
ROC analysis of the CT score for prediction of mortality. N = 22; AUC = 0.833.

The mean CT score of the mortality group (Figure 5) was 50% higher compared to survival group (Figure 6) (*P* = 0.013). In the receiver operating characteristic curve analysis (Figure 7), an optimal cutoff value of a CT score of 21 had a sensitivity of 86% and a specificity of 73% for the prediction of mortality. The area under the receiver operating characteristic curve was 0.833 (95% confidence interval: 0.659, 1). There was no statistically significant difference in the frequency and distribution of the CT findings between the survival and mortality groups.

Table 4 presents the frequency of CT findings, distribution patterns, and CT score of the affected lung parenchyma among 12 patients of the survival group who underwent follow up CT scans at discharge. The mean score of the affected lung parenchyma at discharge was 30% lower than the initial CT examination (*P* = 0.029). There was no statistically significant difference in CT findings and distribution patterns between the initial and follow-up examination.

**Table 4:** Initial and follow-up CT features of 12 patients in survival group.

CT features	Initial CT Scan	CT Scan at Discharge
	N = 12	N = 12
Symptom onset before CT, median(*IQR*), days	5(7–9)	29(26–32)
GGO	1(8)	2(17)
Consolidation	0	0
Both of GGO and consolidation	11(92)	10(83)
CT score, median(*IQR*)	19(11–24)	11(9–16)*
Air bronchogram	8(67)	6(50)
Nodular opacities	1(8)	0
Lymphadenopathy	1(8)	1(8)
Pleural effusions	7(58)	9(75)
Unilateral	4(33)	8(67)
Bilateral	3(25)	1(8)
Anatomic sides involved		
Unilateral	1(8)	2(17)
Bilateral	11(92)	10(83)
Predominant distribution		
Central	0	0
Peripheral	0	2(17)
Both central and peripheral	12(100)	10(83)
Involved zone		
Upper	10(83)	8(67)
Middle	12(100)	11(92)
Lower	12(100)	12(100)

*P<0.05 versus survival group.

Percentages are in parentheses, unless indicated as IQR (interquartile range).

GGO: ground grass opacity.

## Discussion

In this study, patients who were hospitalized with H7N9 virus infection tended to be older. Among all patients, 68% had one or more coexisting medical conditions such as hypertension, cardiac disease, and chronic obstructive pulmonary disease. This finding that H7N9 virus infection appears to attach older adults with coexisting chronic diseases is consistent with prior study [4]. Indeed, a proportion of elderly people have some level of sensitized immune system following exposure to the 1918 virus or related strains that circulated until 1957 [11], and elderly and immunocompromised persons are at increased risk for the development of fulminant influenza virus pneumonia [12]. Of note, 56% (62/111) patients with H7N9 infection provided a clear history and reported close contact with live poultry in previous 14 days before the onset of illness [4]. In our study, however, only 9% (2/22) patients had the same history.

In the present series, frequencies of mechanical ventilation and acute respiratory distress syndrome was higher in mortality group than in survival group, while survival and mortality groups showed no significant differences with respect to patient age, sex, coexisting medical condition, and clinical findings. This is different from H1N1 pneumonia or severe acute respiratory syndrome (SARS). In the previous reports, patients with H1N1 pneumonia or SARS who died were significantly older than those who survived [9], [13]. A possible explanation is the predominance of elderly patients in this outbreak of H7N9 virus infection. However, patients who died had significantly higher frequencies of mechanical ventilation and acute respiratory distress syndrome than those who survived. Acute respiratory distress syndrome is a strong predictor of mortality, which reflects the severity of respiratory failure [14]. Furthermore, for patients with H7N9 virus infection, mechanical ventilation is an important treatment when patients experience respiratory failure [15].

In our study, the overall initial chest radiographic and CT scores were 50% higher in mortality group than in survivors. For the survivors at discharge, their CT score is 30% lower compared with score at admission. Regarding the prediction of mortality, we found an optimal cutoff value of a chest radiographic score of 19 (sensitivity of 71% and specificity of 67%), and a CT score of 21 (sensitivity of 86% and specificity of 73%). The overall radiologic score was based on the severity of air-space disease and its distribution. The more extensive consolidations were observed in the mortality patients than in the survivors. This can be probably explained by the report that main cause of death is hypoxemia [4]. The previous studies stated that patients of viral pneumonias with consolidations on CT have a more severe clinical course than those who present with ground glass opacities [16], [17]. These abnormalities can be pathologically correlated with diffuse alveolar damage [18]. Patients who died tended to have more consolidation and asymmetric disease, while patients of viral pneumonias with bilateral consolidations had a more protracted clinical course [17]. Because H7N9 virus infection is an extremely contagious and potentially fatal disease, risk stratification in our study using the simple scoring method may help triage patient, and may indicate that more aggressive treatment and closely monitoring of disease progression should be applied to the patient with higher overall radiologic scores. However, the efficacy of such approaches to decrease mortality remains to be validated in future study.

Our study demonstrated that a mixture pattern of ground glass opacities and consolidation, air bronchogram, and small pleural effusion were of consistent common radiographic and CT features in both survival and mortality group, distributing in bilateral and central and peripheral locations in the early course of the disease. Our finding of a mixed pattern are consistent with the first report indicating that all three patients had coexisting ground glass opacities and consolidation on radiographs or CT on day 7 after the onset of illness [15], and consistent with a recent report in 111 cases [4]. However, this finding was different from the CT findings in H1N1 influenza A (H1N1) virus-associated pneumonia by Marchiori et al. [19] who noted that ground glass opacity (12/20, 60%) was the most common CT finding and that a mixed pattern (6/20,30%) was the second most common finding 4–9 days after the onset of symptoms. Studies on the CT findings of pneumonia due to H5N1 infection are limited and do not distinguish between ground glass opacity and consolidation [20]. In addition, mediastinal or hilar lymphadenopathy was rare. This finding is similar to the CT appearance of pneumonia caused by H1N1 and H5N1 virus infections, which have been consistently reported in other studies [21]–[23]. A small pleural effusion was observed in 12 cases (55%), including bilateral effusion in 6 patients and unilateral effusion in 6 patients. Pleural effusions are generally considered rare with human influenza A infection [20]. However, these occurred relatively frequently in our patients. A previous study also reported pleural effusions with often severe coexisting consolidation observed in one third of 178 patients infected by H5N1 avian influenza virus, and the effusions were bilateral in nearly all patients during the course of the disease [23]. In addition, a small pleural effusion was previously observed in 5 patients (25%) with H1N1 infection using CT [19].

In this study, we evaluated the prognostic implication of both chest radiographic and CT scores assessed by determining the extent of patchy areas of ground-glass attenuation mixed with consolidation. The chest radiographic and CT scores could help perform risk stratification. In our study, CT attention as HU was −313.2 (±123.8) for ground-glass attenuation, and 11.8 (±79.7) for consolidation areas. We found that the CT attention was helpful to determine ground-glass attenuation and consolidation areas. Using the grading method, the scores at initial chest radiographs and CT images have significance in the prediction of clinical outcomes. Extensive involvement of both lungs was associated with adverse prognosis. Further investigations are warranted to apply this semi-quantitative approach of grading system to calculate or predict ultimate outcome, evaluate disease phenotypes, monitor disease progression, and evaluate treatment response and efficacy for other viral pneumonia or other air space diseases in genetic studies, clinical researches, epidemiological investigations, and clinical trials.

There were several limitations in this study. First, it was retrospective and included only relatively small number of cases. All of the patients in our current study were reported to the CDC from March 2013 to May 2013 and required hospitalization. Therefore, the analysis was biased toward patients with more severe forms of the disease. Second, despite the use of a standardized data-collection form, not all information was collected for all patients. Because none of the mortality patients performed follow-up CT scans, the follow-up CT images included only patients of the survival group with available follow-up CT examination at discharge. Finally, there was a lack of information regarding interobserver agreement, because the study design placed a greater emphasis on the final consensus interpretation rather than independent reading.

In conclusion, we have evaluated the prognostic implication of both chest radiographic and CT score assessed by determining the extent of patchy areas of ground glass attenuation mixed with consolidation in patients with H7N9 infection. High initial chest radiographic or CT score in patients with avian influenza H7N9 pneumonia is associated with mortality, especially in patients with acute respiratory distress syndrome and requiring mechanical ventilation. Since H7N9 virus infection is potentially fatal and highly contagious, risk stratification based on the air-space disease may help triage patients, guide treatment, and monitor disease progression and treatment response.
